# The etiology and prevalence of urinary tract infection and asymptomatic bacteriuria in pregnant women in Iran: a systematic review and Meta-analysis

**DOI:** 10.1186/s12894-019-0454-8

**Published:** 2019-05-30

**Authors:** Milad Azami, Zahra Jaafari, Mansour Masoumi, Masoumeh Shohani, Gholamreza Badfar, Leily Mahmudi, Shamsi Abbasalizadeh

**Affiliations:** 1 Faculty of Medicine, Ilam University of Medical Sciences, Ilam, Iran; 2 Department of Obstetrics & Gynecology, Women’s Reproductive Health Research Center, School of Medicine, Tabriz University of Medical Sciences, Tabriz, Iran; 3 HIV/STI Surveillance Research Center, and WHO Collaborating Center for HIV Surveillance, Institute for Futures Studies in Health, Kerman University of Medical Sciences, Kerman, Iran; 4 Department of Urology, Faculty of Medicine, Ilam University of Medical Sciences, Ilam, Iran; 5 Department of Nursing, Faculty of Nursing and Midwifery, Ilam University of Medical Sciences, Ilam, Iran; 6 Department of Pediatrics, Behbahan Faculty of Medical Sciences, Behbahan, Iran; 7 Faculty of Medicine, Dezful University of Medical Sciences, Dezful, Iran

**Keywords:** Etiology, Urinary tract infection, Asymptomatic bacteriuria, Pregnant women, Iran

## Abstract

**Background:**

Urinary tract infection (UTI) is a common clinical problem in pregnant women. Bacteriuria in pregnancy without antibiotic treatment could result in complications. This study aims to investigate the etiology and prevalence of UTI and asymptomatic bacteriuria (ASB) in pregnant women in Iran.

**Method:**

This meta-analysis follows the Preferred Reporting Items for Systematic Reviews and Meta-Analyses (PRISMA) guidelines. To avoid bias, all steps of the study were carried out independently by two researchers. We conducted a comprehensive search on all the related literature in national databases, including IranDoc, SID, Barakat Knowledge Network System, RICST, Magiran, Iranian National Library and international databases, including Scopus, Embase, Science Direct, PubMed/ Medline, Cochrane Library, Web of Sciences, EBSCO, as well as Google Scholar search engine until June 2018. After considering the inclusion/exclusion criteria and qualitative evaluation, studies were analyzed based on random effects model using Comprehensive Meta-Analysis Software Version 2.

**Results:**

In 31 studies with a sample size of 20,309, the prevalence of ASB in pregnant Iranian women was estimated to be 8.7% (95%CI: 7.2–10.4). The lowest and highest prevalence of ASB were observed in the third trimester (6.1% [95%CI: 2.1–16.4]) and first trimester (11.7% [95%CI: 7.9–16.9]), respectively. Subgroup analysis of the prevalence of ASB based on geographical region (*P* = 0.002) and province (*P* <  0.001) was significant but for the quality of studies (*P* = 0.51) was not significant. In 17 studies including 48,731 pregnant women, the prevalence of UTI was estimated to be 9.8% (95%CI: 7.6–12.5). The test for subgroup differences of prevalence of UTI for province (*P* <  0.001) was significant but for geographical region (*P* = 061) and quality of studies (*P* = 0.11) was not significant. Meta-regression model for the prevalence of UTI and ASB in pregnant women in Iran based on year of the studies was significant (*P* <  0.001). The most common microorganism involved in the etiology of UTI (61.6% [95%CI: 51.6–70.7]) and ASB (63.22% [95%CI: 51.2–73.8]) was *E. coli*.

**Conclusion:**

UTI and ASB are prevalent in pregnant women in Iran. Therefore, UTI screening is essential in pregnant women. The most common microorganism involved in the etiology of UTI and ASB in pregnant women in Iran is *E.coli*.

## Background

Urinary tract infection (UTI) is a common clinical problem that constitutes about 1–6% of medical referrals and includes urinary tract, bladder and kidney infections [1]. UTI may be symptomatic or asymptomatic, while asymptomatic bacteriuria (ASB) is of particular importance due to lack of any symptom [2, 3]. UTI and its related complications cause about 150 million deaths per year around the world [4].

In pregnant women, physiological and anatomical changes in the urinary tract, as well as immune system changes during pregnancy increase the prevalence of ASB and in some cases lead to the symptomatic infection, resulting in serious risks for both mother and fetus. Increasing age, parity, diabetes, sickle cell anemia, history of UTI, urinary tract disorders and immune deficiency may increase the risk of UTI in pregnant women [5–7].

Bacteriuria in pregnancy without antibiotic treatment could result in complications such as preterm labor, pre-eclampsia, hypertension, pyelonephritis, anemia, amnionitis, low birth weight, neonatal deaths (stillbirths), bacteremia and toxic septicemia [8–10]. Treatment of bacteriuria in pregnancy reduces the risk of complications. Therefore, screening for early diagnosis and treatment of bacteriuria in women during pregnancy is necessary to prevent its complications [11].

The overall prevalence of bacteriuria in pregnant Iranian women was reported to be 2–41% [11–54]. Therefore, there is inconsistency in the results of studies. Thus, determining the prevalence of ABS, UTI and the most common pathogenic microorganisms involved in its creation is a valuable diagnostic capability in different countries.

Because of the inconsistency in different reports, reviewing various studies cannot be sufficient to achieve this goal. In systematic reviews, examining all related documents and combining them through meta-analysis provides a more complete picture of the dimensions of a problem [55–57].

This study aims to assess the prevalence of UTI, ASB and pathogens involved in bacteriuria among pregnant women in Iran.

## Methods

### Study protocol

This systematic review and meta-analysis follows the Preferred Reporting Items for Systematic Reviews and Meta-Analyses (PRISMA) guidelines [57]. To avoid bias, all steps of study were carried out independently by two researchers and in case of controversies, the problem was resolved by a third researcher.

### Inclusion and exclusion criteria

Inclusion criteria according to PICO (Evidence-Based Medicine) [58] were as follows [1]: **P**opulation: The epidemiologic studies that investigated UTI, ASB and etiology among pregnant women [2]; **I**ntervention: Urine culture for confirmed UTI and ASB [3]; ** C**omparison: That can show the prevalence of UTI and ASB based on geographical region, province and trimester of pregnancy [4]; **O**utcome: Studies that estimated the UTI, ASB and etiology prevalence in pregnant women.

The exclusion criteria were: 1. Non-random sampling; 2. Non-pregnant Iranian women; 3. Irrelevance with the subject of the research; 4. Incomplete information such as failing to report the prevalence; 5. Qualitative studies; 6. Review articles, case reports and editorials; 7. Duplicates.

### Search strategy and study selection

We conducted a comprehensive search on all English and Persian related literature in national databases, including Iranian Research Institute for Information Science and Technology (IranDoc) (https://irandoc.ac.ir), Scientific Information Database (SID) (http://www.sid.ir/), Barakat Knowledge Network System (http://health.barakatkns.com), Regional Information Center for Science and Technology (RICST) (http://en.ricest.ac.ir/), Magiran (http://www.magiran.com/), Iranian National Library (http://www.nlai.ir/) and international databases, including Scopus, PubMed/ Medline, Science Direct, Cochrane Library, Embase, Web of Sciences, EBSCO, as well as Google Scholar search engine until June 2018. We searched the articles using English MeSH keywords and Persian equivalents: “Pregnant”, “Gestational”, “Pregnancy”, “Prenatal Care”, “Urinary Tract Infection”, “Bacteriuria”, “Iran” and all possible combinations of keywords using “AND” and “OR” operators for English databases. In addition, the manual search was conducted to find more studies by screening the reference list of all articles included in the meta-analysis. PubMed combination search was as follows: (“Pregnant”[Title/Abstract] OR “Pregnancy”[Title/Abstract]) OR “Gestational”[Title/Abstract] OR “Prenatal Care”[Title/Abstract] AND (“Urinary Tract Infection”[Title/Abstract] OR “Bacteriuria”[Title/Abstract] AND “Iran”[Title/Abstract/Affiliation].

It is worth noting that ‘High Sensitive Searching’ was used in databases; in addition, the search was conducted by qualified researchers and experts in the field of database searching (“M. Azami” and “Z. Jaafari”).

### Quality assessment

Authors assessed the quality of studies according to the modified Newcastle Ottawa Scale (NOS) for cross-sectional studies [59], which includes eight sections, and evaluated the selected articles from the selection, comparability, exposure assessment, and outcome. Points of 0–5, 6–7 and 8–10 were considered as low quality, moderate quality and high quality, respectively. A minimum score of 6 was considered as a criterion to include an article.

### Data extraction

The checklist was designed based on goals. This checklist included: authors, place, province, region, year of publication, year of study, study design, mean age, sample size, prevalence of UTI, ASB and microorganisms involved in bacteriuria. Sample size and prevalence of UTI and ASB for the first, second and third trimester of pregnancy were independently extracted by two researchers.

### Statistical analysis

Binomial distribution formula was used to estimate the standard error for the prevalence of UTI, ASB and pathogens involved in bacteriuria. The heterogeneity of the studies was assessed using Cochran’s Q test and I^2^ index, and interpreted as follows: 0–24% may not be important, 25–49% indicates moderate heterogeneity, 50–75% indicates substantial heterogeneity and over 75% indicates considerable heterogeneity [60]. To combine data in high heterogeneity, we used the random effects model. To explore the potential sources of heterogeneity, subgroup analysis was preformed based on geographical region, province and trimester of pregnancy [61, 62]. We used the meta-regression model for the prevalence of UTI and ASB according to year of the study. Publication bias was measured by reviewing the funnel plots and through Begg and Egger’s tests. Meta-analysis of data was performed using Comprehensive Meta-Analysis Software Version 2 and the significance level was considered less than 0.05.

## Results

### Search results

**Fig. 1:**
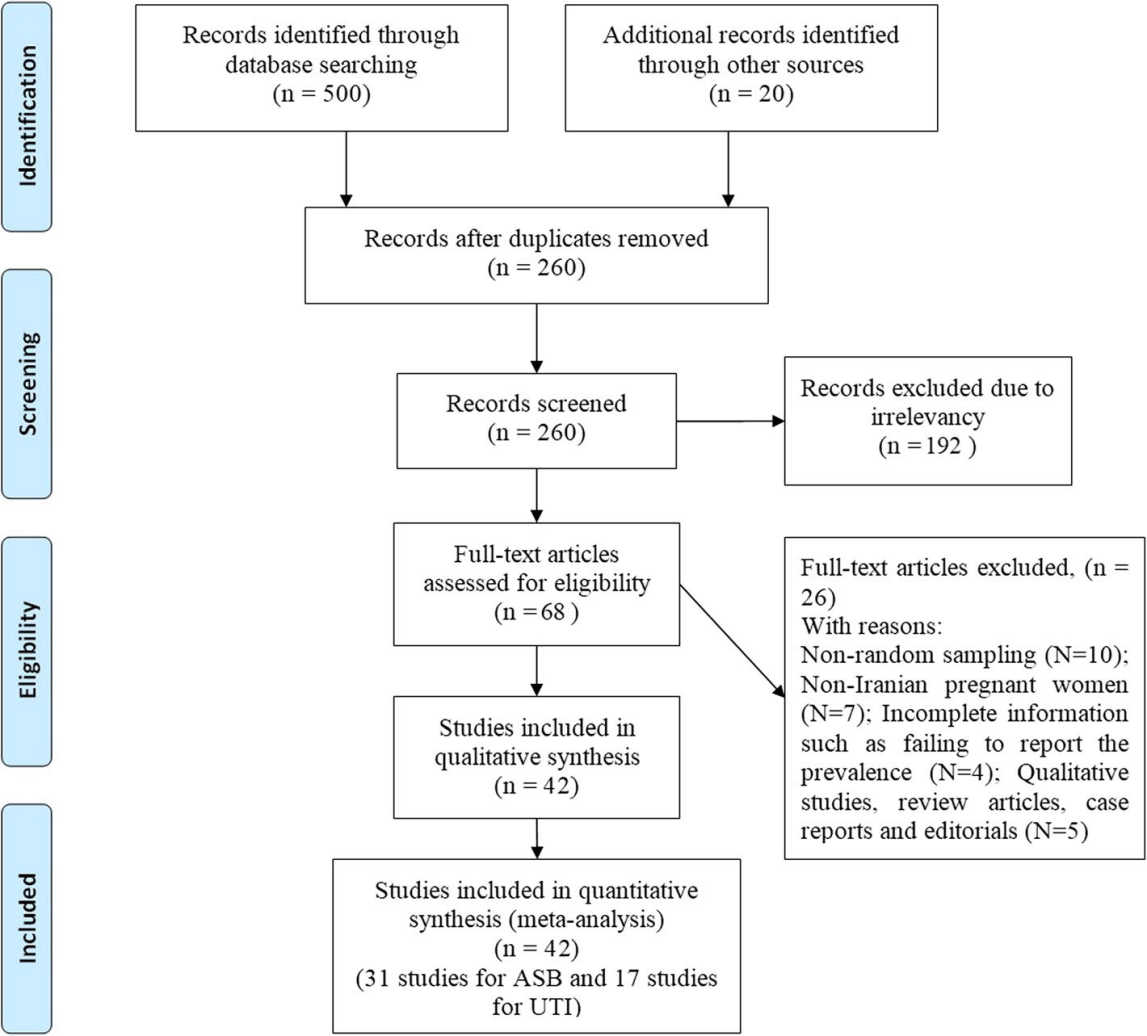
A flow diagram following the PRISMA template

In the systematic review, 520 potentially relevant articles were identified, and after screening the titles and abstracts, 260 studies were excluded because of being duplicate, and the full text of 260 possibly related articles was studied. After the evaluation of exclusion/inclusion criteria and the quality of articles, 42 eligible studies, published from 1995 to 2015, were included in meta-analysis (Fig. 1).

### Study characteristics

42 eligible articles (17 studies for UTI and 31 studies for ASB) including 67,776 pregnant women were investigated. Mean and standard deviation (SD) for age was 26.47 ± 5.47 years. Other study characteristics are shown in Table 1.

**Table 1:** Characteristics of studies on asymptomatic bacteriuria and urinary tract infections among pregnant Iranian women

Ref	First author, published year	Place	Year of study	Sample size	(Mean±SD^a^) age	Prevalence of ASB^b^	Prevalence of UTI^c^	Test	Most common microorganisms
[12]	Vejdani MH, 1998	Tabriz	1995	950		10.5		Culture	E.coli^d^
[13]	Farajzadegan Z, 2008	Isfahan	2008	100	25.1 ± 3.6	2		Culture	
[14]	Shirazi MH, 2007	Hamadan	2007	377		10.1		Culture	E.coli
[15]	Safari M, 2008	Yasuj	2006	715	25 ± 5.3		6	Culture	
[16]	Mobsheri E, 2002	Gorgan	2000	900		3.7		Culture	E.coli
[17]	Kameli M, 2013	Torbat Haidaria	2013	1250		10		Culture	*Staphylococcus epidermidis*
[18]	Soofizadeh N, 2012	Sanandaj	2009	1500	28.4 ± 6.1	7.6		Culture	–
[19]	Azizzadeh Sh, 1999	Tehran	1997	547		12.7		Culture	E.coli
[20]	Aaron H, 2008	Kerman	2007	323		24.1		Culture	–
[21]	Aghaee alamouti M, 2010	Tehran	2009	826		8.7		Culture	–
[22]	Yousofzadeh Sh, 1995	Kashan	1995	400		4.7	7.5	Culture	–
[23]	Necohesh L, 2005	Ghods	2004	100		14		Culture	E.coli
[24]	Namazi A, 2012	Guilan	2008	710	27.48 ± 6.0	21.1		Culture	–
[25]	Shojaee H, 2000	Shahrekord	1999	864		4.3	8.8	Culture	E.coli
[26]	Zarganj Fard A, 2000	Arak	2000	1736		6.3		Culture	E.coli
[27]	Khorshidi A, 1997	Kashan	1996	350	24 ± 5.5	5.4		Culture	E.coli
[28]	Hazhir S, 2007	Tabriz	2007	1100		6.1		Culture	–
[29]	Motaghi M, 2012	Mashhad	2008	150		10.7		Culture	E.coli
[30]	Dadkhah F, 2011	Tehran	2010	1246		9		Culture	–
[31]	Kalantar E, 2008	Sanandaj	2008	1505	28.4 ± 4.6	8.9		Culture	E.coli
[32]	Kasraeian M, 2009	Shiraz	2007	389	26.3 ± 4.2	5.1		Culture	E.coli
[33]	Jazayeri Moghadas A, 2009	Semnan	2007	297		3.3		Culture	E.coli
[34]	Alavi-Naini R, 2003	Zahedan	2002	490		14.9		Culture	E.coli
[34]	Alavi-Naini R, 2003	Zahedan	2002	478		10.5		Culture	E.coli
[34]	Alavi-Naini R, 2003	Zahedan	2002	463		16.2		Culture	E.coli
[35]	Rahmani E, 2012	Kermanshah	2011	500	27.4 ± 5.8		19.8	Culture	–
[36]	Norouzzadeh M, 1997	Karaj	1997	300			22.7	Culture	E.coli
[37]	Mardanian F, 2004	Isfahan	2004	543		10.4		Culture	–
[37]	Mardanian F, 2004	Isfahan	2004	911		9.9		Culture	–
[37]	Mardanian F, 2004	Isfahan	2004	891		2.0		Culture	–
[38]	Fakhimi L, 2002	Zanjan	2002	1012			13	Culture	–
[39]	Azizi A, 2015	Sanqor	2001	3157	26.5 ± 5.5		5.8	Culture	–
[40]	Jalali M, 2014	Karaj	2013	180	26 ± 5.8		19.8	Culture	E.coli
[41]	Zakeri Hamidi M, 2006	Mazandaran	2004	300			8.33	Culture	–
[42]	Shahhosseini Z, 2012	Sari	2009	428	24.5 ± 4.8		2.8	Culture	–
[43]	Rahimkhani M, 2008	Tehran	2012	86	26.8 ± 5.5	29.1		Culture	Staphylococcus epidermidis
[44]	Golestan M, 2011	Yazd	2008	5897			6.98	Culture	–
[45]	Rahmanian M, 2014	Semnan	2012	160	27.8 ± 5.4	5.6		Culture	–
[46]	Shams MR, 2000	Tehran	1996	205		6.8		Culture	–
[47]	Alijahan R, 2014	Ardabil	2011	2496			9.7	Culture	E.coli
[48]	Saffar MJ, 2008	Sari	2002	5600			12.6	Culture	E.coli
[49]	Soleimani Zadeh L, 2004	Bam	2001	850	25.5 ± 6.6		12.3	Culture	–
[50]	Modars Sh, 1998	Tehran	1996	462		10.8		Culture	E.coli
[51]	Sharemi H, 2013	Rasht	2012	330	27.9 ± 5.8		23.6	Culture	–
[52]	Sohrabi D, 2011	Zanjan	2007	3102			5.83	Culture	–
[53]	Amiri M, 2015	Dezful	2012	22,600		10.5	5	Culture	E.coli

^a^Standard deviation; ^b^ Asymptomatic bacteriuria; ^c^ Urinary tract infections; ^d^*Escherichia coli*

*Some studies estimated the prevalence of UTI or ASB for more than 1 year and also regions. Each data was considered separately because of assessing the slope of prevalence in the years and estimating which region is the highest or lowest

### Total prevalence of ASB and sensitivity analysis

**Fig. 2:**
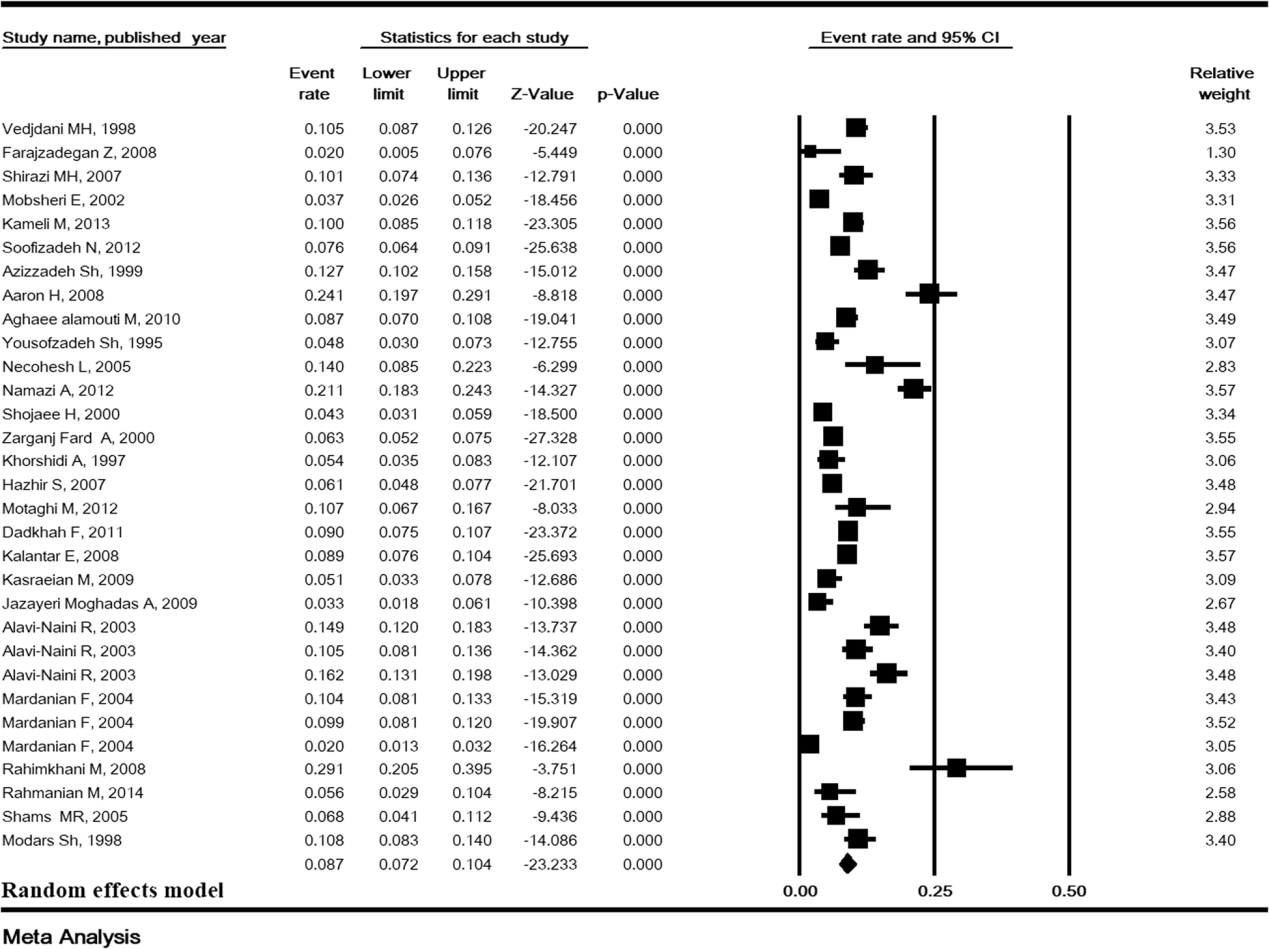
Prevalence of asymptomatic bacteriuria in pregnant women in Iran

**Fig. 3:**
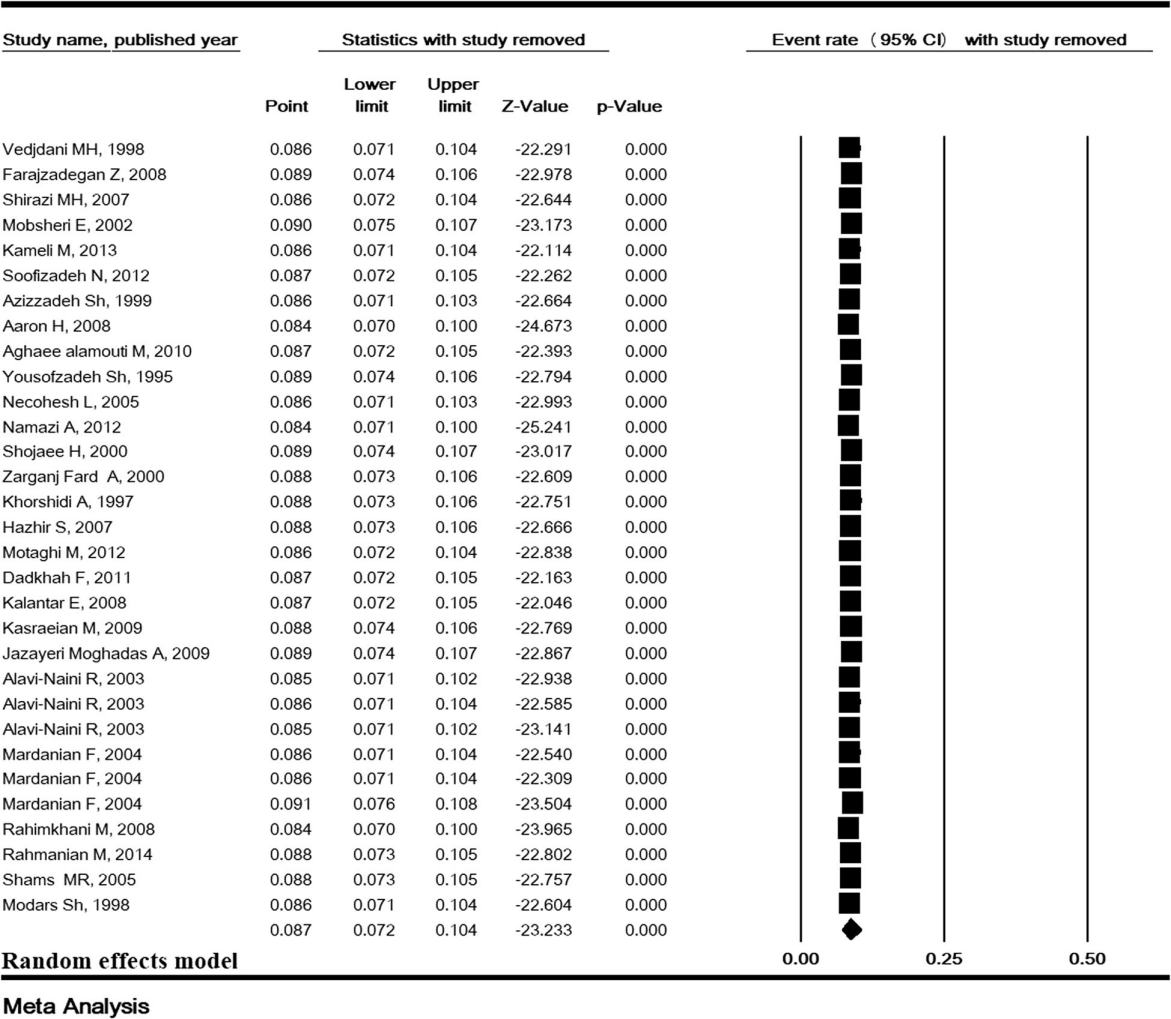
Sensitivity analysis for the prevalence of asymptomatic bacteriuria in pregnant women in Iran

The heterogeneity rate for the prevalence of ASB was high (I^2^ = 93.38, *P* <  0.001). In 31 studies with a sample size of 20,309, the prevalence of ASB in pregnant Iranian women was estimated to be 8.7% (95% CI:7.2–10.4) (Fig. 2). The lowest and highest prevalence of ASB were 2 and 29.1% in the studies of Farajzadegan [13] and Rahimkhani [43], respectively (Fig. 2). Sensitivity analysis for the prevalence of ASB in Fig. 3 shows that after removing a study at a time, the result is still robust.

### Subgroup analysis of the prevalence of ASB based on geographical region and province

The lowest prevalence of ASB in pregnant women was estimated to be in the South of Iran (5.1% [95% CI: 3.3–7.8]) and Golestan province (3.7% [95% CI: 2.6–5.2]). The highest prevalence of ASB in pregnant women was estimated to be in the East of Iran (13.9% [95% CI: 10.3–18.6]) and Kerman province (24.1% [95% CI: 19.7–29.1]). The test for subgroup differences for geographical region (*P* = 0.002) and province (*P* <  0.001) was significant (Table 2).

**Table 2:** subgroup analysis for the prevalence of asymptomatic bacteriuria and urinary tract infections in pregnant women in Iran

Variable	Asymptomatic bacteriuria						Urinary tract Infection					
	Studies (N^a^)	Sample (N)	Heterogeneity		95%CI^b^	Pooled prevale nce (%)	Studies (N)	Sample (N)	Heterogeneity		95%CI	Pooled prevalence (%)
			I^2^	*P*-Value					I^2^	*P*-Value		
Region												
Center	17	9724	90.01	< 0.001	5.8–9.4	7.4	6	11,575	96.42	< 0.001	6.7–13.8	9.7
East	6	3154	90.19	< 0.001	10.3–18.6	13.9	–	–	–	–	–	–
North	3	2560	98.08	< 0.001	3.9–22.1	9.7	6	9334	94.77	< 0.001	8.2–15.6	11.4
South	1	389	–	–	3.3–7.8	5.1	3	24,165	97.55	< 0.001	3.9–13.1	7.2
West	4	4482	68.68	0.023	6.6–9.6	8.0	2	3657	99.04	< 0.001	3.1–32.4	11.0
Test for subgroup differences: Q = 17.07, df(Q) = 4, *P* = 0.002							Test for subgroup differences: Q = 1.77, df(Q) = 3, *P* = 0.61					
Province												
East Azarbaijan	2	2050	92.25	< 0.001	4.7–13.5	8.1	1	400	–	–	5.3–10.5	7.5
Isfahan	6	3195	91.29	< 0.001	3.1–8.8	5.2	–	–	–	–	–	–
Hamedan	1	377	–	–	7.4–13.6	10.1	–	–	–	–	–	–
Golestan	1	900	–	–	2.6–5.2	3.7	–	–	–	–	–	–
Khorasan Razavi	2	1400	0	0.78	8.6–11.8	10.1	–	–	–	–	–	–
Kurdistan	2	3005	40.25	0.19	7.1–9.6	8.3	–	–	–	–	–	–
Tehran	7	3472	85.23	< 0.001	8.8–15.3	11.7	–	–	–	–	–	–
Kerman	1	323	–	–	19.7–29.1	24.1	1	850	–	–	10.3–14.7	12.3
Chaharmahal and Bakhtiari	1	864	–	–	4.7–13.5	8.1	1	864	–	–	7.1–10.9	8.8
Markazi	1	1736	–	–	5.2–7.5	6.3	–	–	–	–	–	–
Fars	1	389	–	–	3.3–7.8	5.1	–	–	–	–	–	–
Semnan	2	457	26.81	0.24	2.5–7.1	4.3	–	–	–	–	–	–
Guilan	1	710	–	–	18.3–24.3	21.1	1	330	–	–	19.3–28.5	23.6
Sistan and Baluchestan	3	1431	71.41	0.03	10.8–17.5	13.8	–	–	–	–	–	–
Kohgiloyeh and Boyerahmad	–	–	–	–	–	–	1	715	–	–	4.5–8.0	6.0
Kermanshah	–	–	–	–	–	–	2	3657	99.04	< 0.001	3.1–32.4	11.0
Alborz	–	–	–	–	–	–	2	480	0	0.45	18.2–25.6	21.6
Zanjan	–	–	–	–	–	–	2	4114	98.11	< 0.001	3.9–18.5	8.8
Mazandaran	–	–	–	–	–	–	3	6328	94.07	< 0.001	3.2–14.6	7.0
Ardebil	–	–	–	–	–	–	1	2496	–	–	8.6–10.9	9.7
Yazd	–	–	–	–	–	–	1	5897	–	–	6.4–7.7	7.0
Khuzestan	–	–	–	–	–	–	1	22,600	–	–	4.7–5.3	5.0
Test for subgroup differences: Q = 289.16, df(Q) = 13, *P* < 0.001							Test for subgroup differences: Q = 491.83, df(Q) = 11, *P* < 0.001					
Quality												
High	19	12,500	94.19	< 0.001	6.4–10.6	8.3	11	45,355	98.42	< 0.001	6.4–11.5	8.6
Moderate	12	7809	92.25	< 0.001	7.1–12.1	9.3	6	3376	93.11	< 0.001	8.7–17.6	12.5
Test for subgroup differences: Q = 0.42, df(Q) = 1, P = 0.51							Test for subgroup differences: Q = 2.45, df(Q) = 1, P = 0.11					

^a^Number; ^b^ Confidence interval

### Subgroup analysis of the prevalence of ASB based on quality of studies

The prevalence of ASB among pregnant women in terms of quality of studies based on NOS checklist was estimated to be 9.3% [95% CI: 7.1–12.1]) and 8.3% [95% CI: 6.4–10.6]) in moderate-quality and high-quality studies, respectively. No significant difference was found (*P* = 0.51) (Table 2).

### The prevalence of ASB based on trimester of pregnancy

**Fig. 4:**
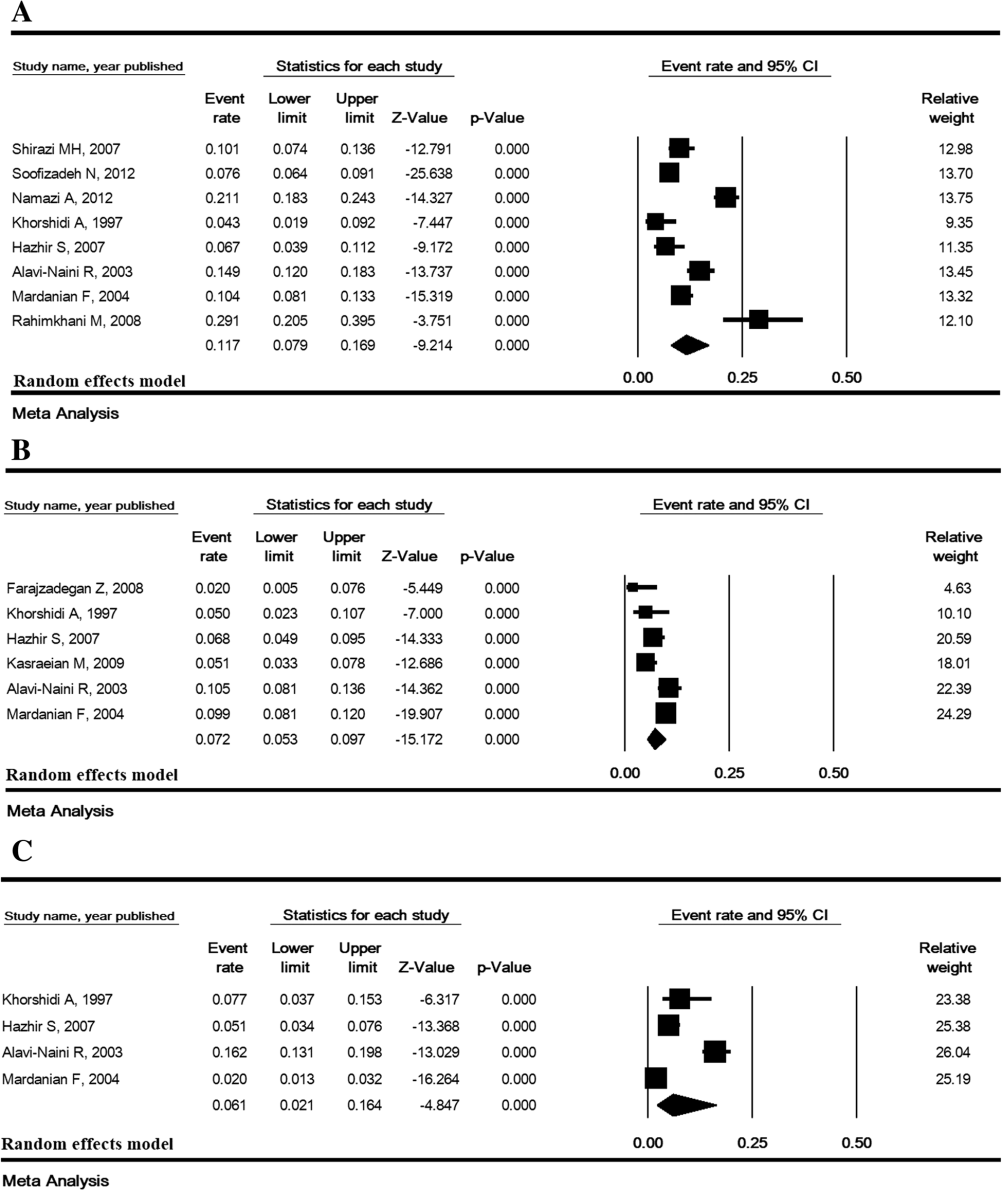
Prevalence of asymptomatic bacteriuria in the first (**a**), second (**b**) and third (**c**) trimesters in Iran

The lowest and highest prevalence of ASB were estimated in the third trimester (6.1% [95% CI: 2.1–16.4]) and first trimester (11.7% [95% CI: 7.9–16.9]), respectively (Fig. 4).

### Prevalence of UTI in pregnant women

**Fig. 5:**
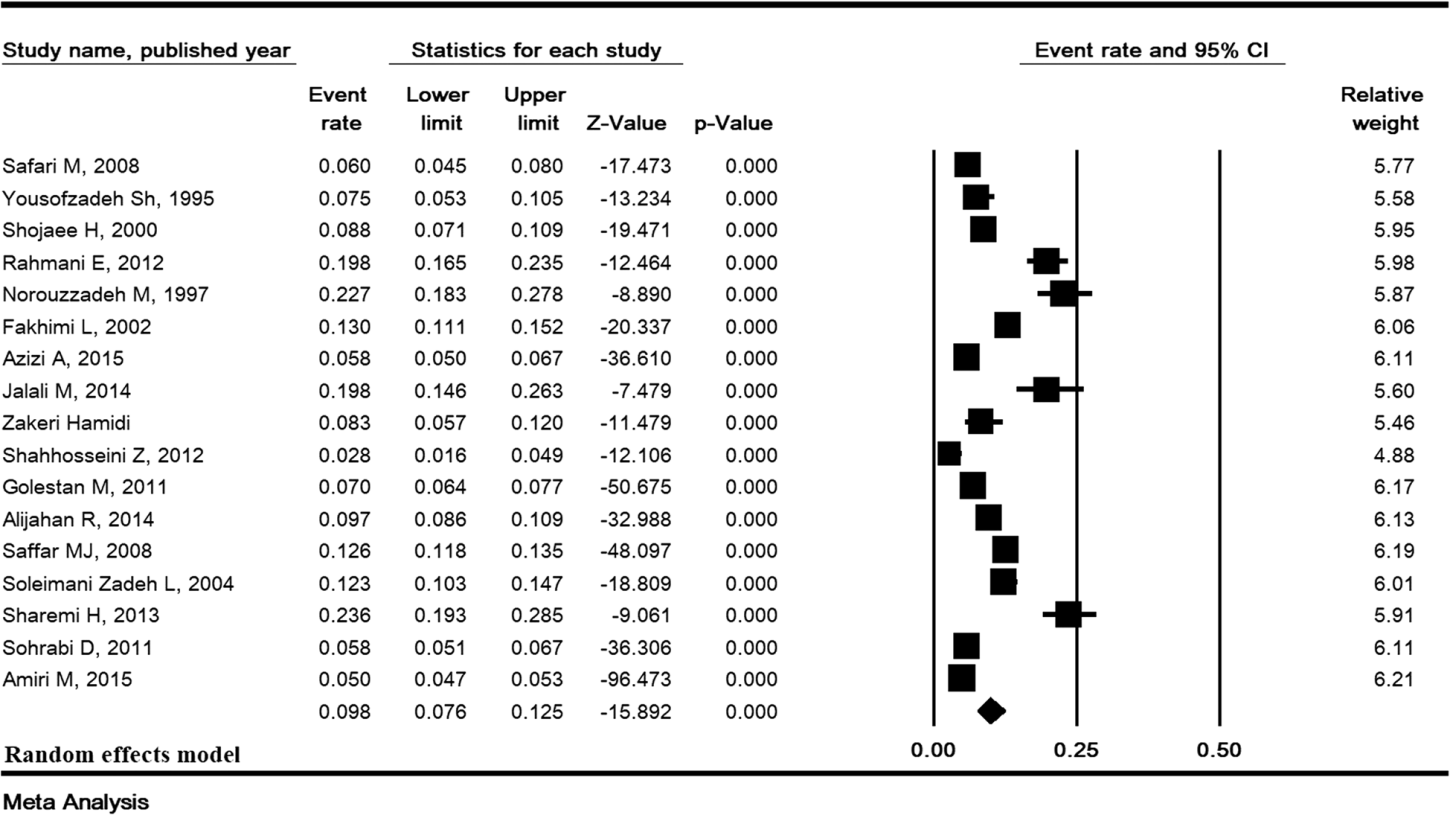
Prevalence of urinary tract infection in pregnant women in Iran

**Fig. 6:**
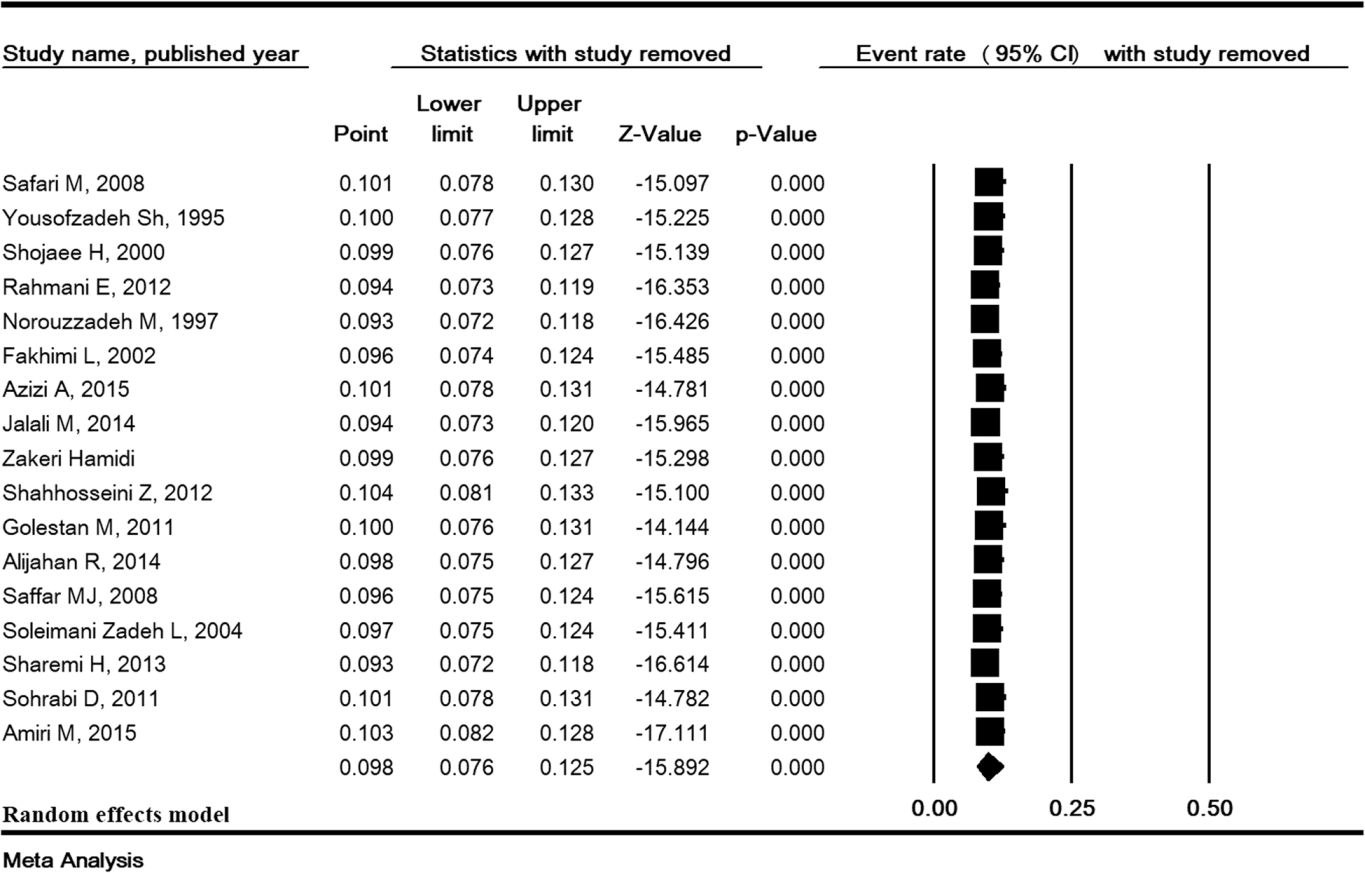
Sensitivity analysis for the prevalence of urinary tract infection in pregnant women in Iran

The heterogeneity rate for the prevalence of UTI was high (I^2^ = 98.12%, *P* <  0.001). In 17 studies including 48,731 pregnant Iranian women, the prevalence of UTI was estimated to be 9.8% (95% CI: 7.6–12.5). The lowest prevalence was in the study of Shahhosseini (2.8%) and the highest prevalence was in the study of Sharemi (23.6%) (Fig. 5). Sensitivity analysis by removing a study at a time showed that the result for the prevalence of UTI was robust (Fig. 6).

### Subgroup analysis of the prevalence of UTI based on geographical region and province

The prevalence of UTI among pregnant women in the South of Iran (7.2% [95% CI: 3.9–13.1]) and Khuzestan province (5% [95% CI: 4.7–5.3]) were the lowest and in the North of Iran (11.4% [95% CI: 8.2–15.6]) and Alborz province (21.6% [95% CI: 18.2–25.6]) were the highest. The test for subgroup differences for geographical region (*P* = 061) was not significant but for province (*P* <  0.001) was significant (Table 2).

### Subgroup analysis of the prevalence of UTI based on quality of studies

The prevalence of UTI among pregnant women in terms of quality of studies based on NOS checklist was estimated to be 12.5% [95% CI: 8.7–17.6]) and 8.6% [95% CI: 6.4–11.5]) in moderate-quality and high-quality studies, respectively. No significant difference was found (*P* = 0.11) (Table 2).

### Total prevalence of UTI and ASB with omission of high prevalence reports

**Fig. 7:**
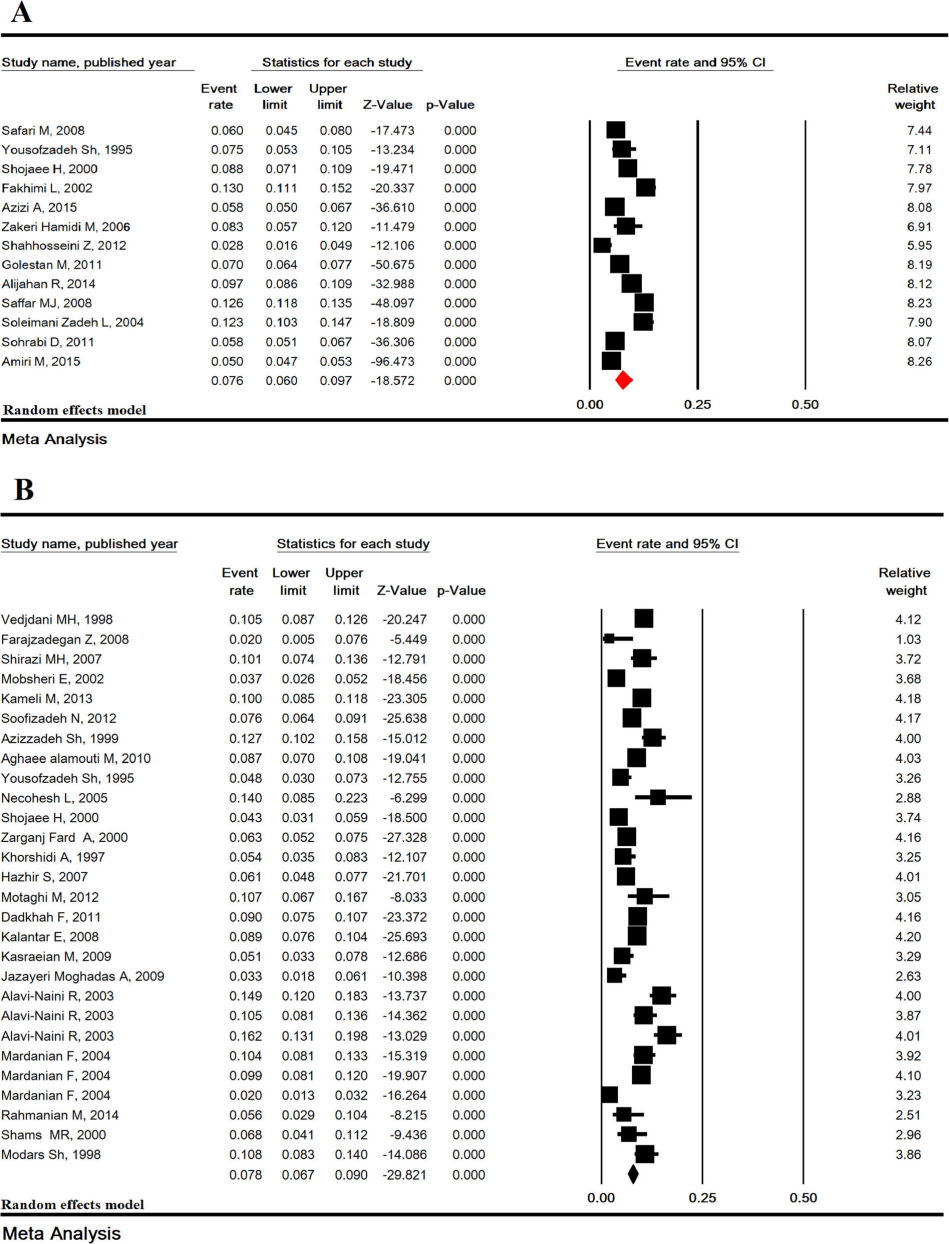
Prevalence of urinary tract infection (**a**) and asymptomatic bacteriuria (**b**) in pregnant women in Iran with deleted high prevalence reports (4 studies for prevalence of UTI: Rahmani, Norouzzadeh, Jalali and Sharemi and 3 studies for ASB: Namazi, Rahimkhani and Aaron)

Four studies (Rahmani [35], Norouzzadeh [36], Jalali [40] and Sharemi [51]) for prevalence of UTI and three studies (Namazi [24], Aaron [20] and Rahimkhani [43]) for prevalence of ASB reported high prevalence. After omitting these studies, the prevalence of UTI and ASB was estimated to be 7.6% (95% CI: 6.0-9.7) and 7.8% (95% CI: 6.7–9.0), respectively (Fig. 7).

### Meta-regression

**Fig. 8:**
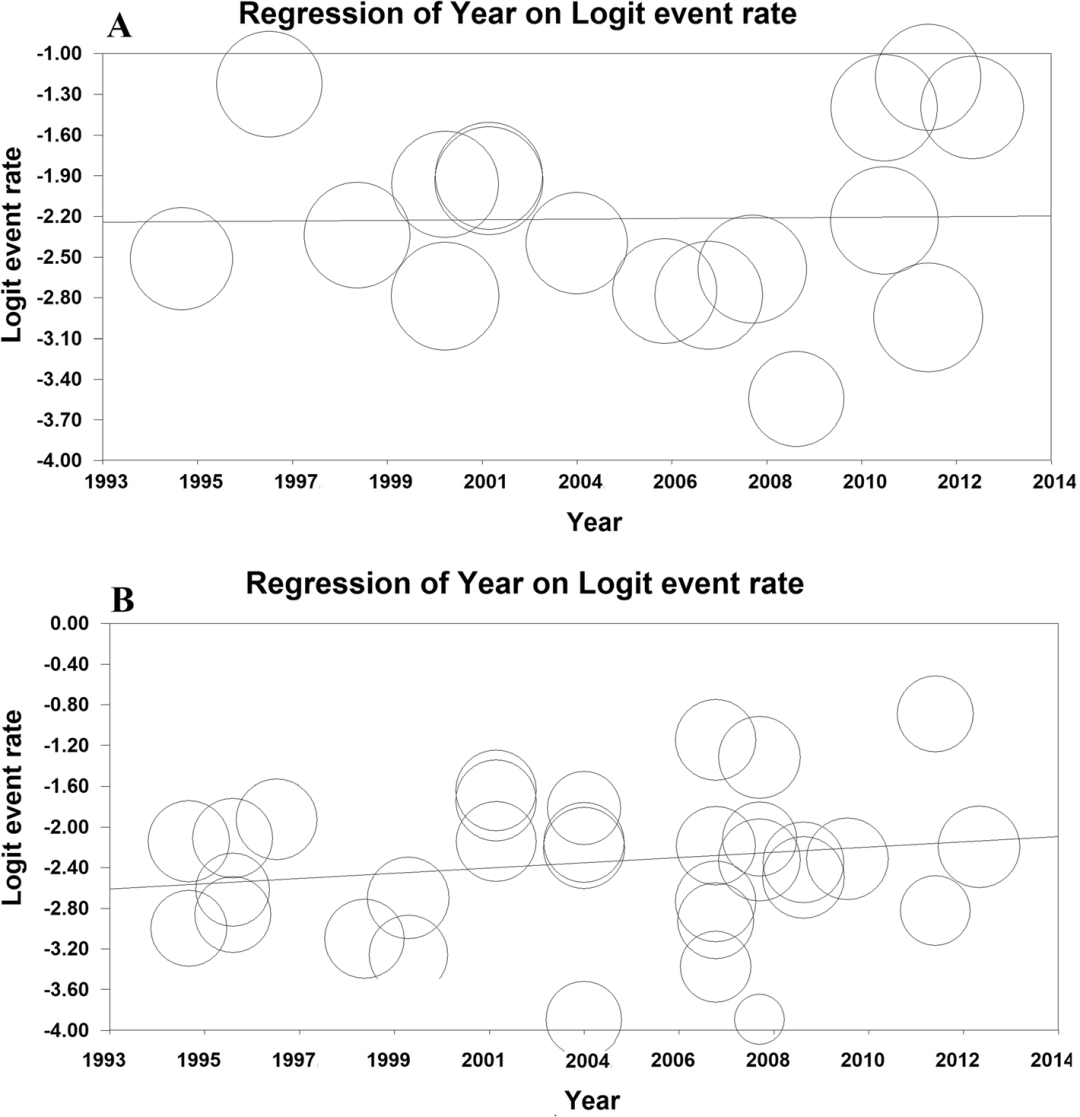
Meta-regression model for the prevalence of urinary tract infection (**a**) and asymptomatic bacteriuria (**b**) in pregnant women in Iran based on year of the studies

Meta-regression model for the prevalence of UTI and ASB in pregnant women in Iran based on year of the studies was significant (*P* <  0.001 for UTI and *P* <  0.001 for ASB) (Fig. 8).

### The prevalence of microorganisms

The most common microorganism involved in the etiology of UTI (61.6% [95% CI: 51.6–70.7]) and ASB (63.22% [95% CI: 51.2–73.8]) was *E. coli*. The lowest prevalence was Proteus with 2.6% (95% CI: 1.9–3.4) for UTI and 3.6% (95%CI: 2.0–6.3) for ASB (Table 3).

**Table 3:** The prevalence of Micro-Organisms in asymptomatic bacteriuria and urinary tract infections among Pregnant Iranian women

Micro-Organisms	Asymptomatic bacteriuria							Urinary tract Infection						
	Studies (N^a^)	Sample (N)	Heterogeneity		Pooled prevalence (%) [95%CI^b^]	Meta-regression		Studies (N)	Sample (N)	Heterogeneity		Pooled prevalence (%) [95%CI]	Meta-regression	
			I^2^	*P*-Value		Trend	*P*-Value			I^2^	*P*-Value		Trend	*P*-Value
*Escherichia coli*	18	1037	91.03	< 0.001	63.2 [51.2–73.8]	DES^c^	< 0.001	6	2255	93.34	< 0.001	61.6 [51.6–70.7]	ASC^c^	0.088
Staphylococcus	15	902	88.09	< 0.001	19.6 [12.7–29.2]	ASC^d^	< 0.001	5	2187	58.99	0.045	9.9 [7.7–12.6]	DES^d^	< 0.001
Klebsiella	12	783	42.86	0.057	6.3 [4.2–9.4]	DES	0.232	3	1448	90.05	< 0.001	13.9 [7.3–25.0]	DES	0.094
Streptococcus	3	145	50.91	0.130	5.2 [1.6–15.9]	ASC	0.046	–	–	–	–	–	–	–
Enterococcus	5	336	24.33	0.259	3.8 [1.8–7.9]	DES	0.165	–	–	–	–	–	–	–
Enterobacter	8	608	29.33	0.194	6.4 [4.1–9.7]	ASC	0.578	2	776	61.51	0.107	7.6 [4.3–13.1]	–	–
Proteus	6	356	0	0.766	3.6 [2.0–6.3]	ASC	0.383	3	1871	0	0.84	2.6 [1.9–3.4]	ASC	0.820

^a^Number; ^b^ Confidence interval; ^c^ descending; ^d^ ascending

## Discussion

The results of this meta-analysis indicated that the prevalence of ASB and UTI in pregnant Iranian women was 8.7 and 9.8%, respectively. In subgroup analysis, geographic region, province and year of the studies can be a cause of heterogeneity between studies. The most common microorganism involved in the etiology of ASB and UTI in pregnant women in Iran was *E. coli* (63.2 and 61.6%, respectively), while meta-regression model based on year of the studies for *E. coli* (for ASB) had a significantly decreasing trend. The healthcare structure for pregnant women is a state funded program running in Iran. This program included urine testing (urine culture test and urine analysis) in the first prenatal visit [12–15].

Prevalence of bacteriuria in pregnancy is affected by several factors such as multiple pregnancies, age, previous history of UTI, diabetes, urinary tract anatomic abnormalities, lack of personal hygiene and socioeconomic status [63, 64]. In a systematic review conducted in Iran in 2015, ASB prevalence in pregnant women was reported to be 13% (95% CI: 9–7) [65] after combining 20 articles (sample size: 15,108). In the present meta-analysis combining 31 studies with a sample size of 20,309 Iranian pregnant women, the prevalence of ASB was 8.7% (95% CI: 7.2–10.4). The strengths of this study compared to previous published meta-analyses include bigger sample size, the use of cross-sectional studies, excluding studies with a non-randomized sample [66, 67] and removal of the duplicate articles that published the results more than once [14, 16, 68, 69]. Each of the suggested factors can affect the final evaluation and accuracy of the prevalence while this was not considered in the previous meta-analyses [65].

The prevalence of ASB in pregnant Iranian women based on trimester of pregnancy shows that the highest prevalence occurs in the first trimester of pregnancy (11.7% [95% CI: 7.9–16.9]). Given that screening for UTI is done before the pregnancy and at 6–10 weeks of gestation in Iran, lack of care before pregnancy may increase the risk of UTI in pregnant women in the first trimester. However, trimester of pregnancy can be one of the causes of diverse prevalence of ASB in Iranian studies (*P* = 0.02).

There seem to be a geographical variation in the ASB prevalence, and we could not find the causes, but a possible reason might include differences in race (there is much racial diversity in Iran), socioeconomic factors, education, quality health care and women’s health services communities [70, 71].

The prevalence of ASB among pregnant women in other countries, including India (7.3%), Nigeria (24.7–45.3%), Nepal (8.7%) Bangladesh (10.2%) and Ethiopia (21.2%) was reported to be different [71–75].

The prevalence of UTI among young women is about 1–3% [76]. The results of this meta-analysis showed that the prevalence of UTI in pregnant Iranian women is high. Pregnant women prone to UTI are at risk for prematurity, preterm delivery, low birth weight, hypertension/pre-eclampsia, anemia, maternal and perinatal death associated with amnionitis [75, 76].

Studies show that the higher the education level, the lower the frequency of this problem. Hence, the need for education and awareness of pregnant women, especially in those with a lower education level, is necessary [18–20].

A common organism of ASB in pregnant women in Iran was *E.coli* (63.2%). In other studies, the most common organism of ASB in women was *E.coli* [77]. *E.coli* is the underlying cause of ASB in 77% of sexually active young American women [78], 72% of girls of school age [79], and 65–84% of pregnant women [80–83].

*E. coli* strains isolated from healthy women without symptoms may have a lower frequency of virulence factors, such as adhesions, specific lipopolysaccharide, toxins, mobility factors, and other proteins compared to strains isolated from symptomatic urinary tract infection [84, 85].

The aim of ASB treatment is to maintain sterile urine without causing toxicity in mother or fetus during pregnancy [82]. However, the best way to achieve this is not clear yet. In low-income countries, the situation is worse due to lack of information about the resistance to drugs used for UTI in pregnancy, drug costs and lack of access to information regarding the safety and efficacy of newer antibiotics [86].

In Iran, drug resistance in antibiotics used for UTI is a controversial topic. Different levels of antibiotic resistance and sensitivity has been reported in different studies. For example, in a study by Enaiat et al., high resistance of nalidixic acid, tetracycline and co-trimoxazole and low sensitivity of ampicillin, gentamicin and amikacin to *E. coli* has been reported in regard with bacteriuria in pregnant women [87]. However, in the study of Saffar, *E.coli* was highly sensitive to gentamicin and amikacin and less sensitive to trimethoprim-sulfamethoxazole and ampicillin. Therefore, considering the importance of empiric therapy for physicians, conducting a systematic review and meta-analysis to determine patterns of microbial resistance to drugs against UTI in Iran seems necessary.

### Limitations

1. Lack of “AND” and “OR” operators support for a combined search in national databases.
2. Failure to investigate the prevalence of UTI based on trimester of pregnancy due to the limited number of studies.
3. Failure to investigate the prevalence of UTI based on factors such as multiple pregnancies, age, previous history of UTI, diabetes, anatomical abnormalities of urinary tract, lack of personal hygiene and socioeconomic status

## Conclusion

UTI and ASB are highly common in pregnant women in Iran and the most common type of UTI is ASB. Therefore, it is recommended that urine culture be conducted as a part of routine tests for pregnant women. Moreover, pregnant women need to be provided with complete information about UTI complications during their pregnancy. The most common microorganisms involved in the etiology of ASB and UTI in pregnant women in Iran are *E.coli* and *Staphylococcus*. Since women and mothers’ health is the foundation of the family and the public health, making better management decisions for prevention, screening and treatment of this problem is recommended.

## Abbreviations

ASB: Asymptomatic Bacteriuria
IranDoc: Iranian Research Institute for Information Science and Technology
PRISMA: Preferred Reporting Items for Systematic Reviews and Meta-Analyses
RICST: Regional Information Center for Science and Technology
SID: Scientific Information Database
UTI: Urinary tract infection
